# Treatment and Destruction of the Pulp

**Published:** 1892-04

**Authors:** Frank French

**Affiliations:** Rochester.


ARTICLE VI.

### TREATMENT AND DESTRUCTION OF THE PULP.

BY DR. FRANK FRENCH, ROCHESTER.

Read at the Union Meeting of the Fifth, Sixth, Seventh and Eighth District Dental Societies of the State of New York, held in Buffalo, October, 1891.

Some years ago, at a meeting of the Dental Society of the State of New York, there was present one of those meteoric interrogation points, who have been heard from in all ages, for we even find them mentioned in Holy Wirt, as follows: "Verily, knowledge shall perish with them," Tim. 10: 16. Accosting one of the older practitioners, he said: "Well, Dr. Blank, I suppose you save all nerves?" The reply was, "Yes, certainly." "Will you tell me how you save them?" "Yes; some of them I dry, and some I preserve in alcohol." While I do not by any means advocate the wholesale destruction of the pulp of the tooth, for I believe in saving it alive if possible, I think most practitioners of fifteen years' standing will agree that those which have been dried and saved in alcohol are far more satisfactory, both to patient and dentist, as a rule, than those which have been capped, excepting, of course, those where the conditions were entirely favorable. Various methods have been used to destroy the pulp, the most primitive being to heat a fine instrument or wire red hot and thrust it as far into the pulp canal as possible. A modification of this treatment has for several years been practiced by the lightning bridge and crown workers, who taper a piece of hickory or orange wood to a very fine point, and, with a mallet, drive it into the pulp canal. Both these treatments are very efficacious, and absolutely without pain——to the dentist. A very interesting article might be written, giving a history of the different means employed to destroy the pulp, but it is not within the scope of this paper.

We all of us have cases where, for a variety of reasons, it is advisable to destroy the pulp. How can this be done with almost absolute certainty, and with very little, if any, discomfort to the patient? Under the head of "Incidents of Office Practice," in our District, as well as in our City Society, it has been mentioned several times that difficulty had been experienced in destroying the pulp, even with several applications of arsenial paste, and that much discomfort and pain to the patient had followed. During a practice extending over many years, it very rarely happens—not more than once or twice a year—that I have to make a second application to destroy a pulp, and almost as rarely is it attended with pain. Making this statement to several of the younger members of our society, I was asked to "write it up" for this meeting. I do not lay claim to anything new or original, but do know that I save my patients much sufferings, and for this mercy they are truly grateful.

Any process of destroying the pulp, other than that of immediate extirpation, takes time. The action of the various medicaments when applied to it are as follows: Irritation, congestion, inflammation, death. The pulp, as we all know, while of fair size in the body of the tooth, gradually grows smaller as it approaches the apex, until where it passes through the apical foramen it is scarcely more than a hair in size. The effect of the article used causes, as I have said, irritation, congestion, inflammation. There is a determination of blood to those parts, the vessels become congested and inflamed, the opening at the foramen is so small that it is closed by the enlargement of the vessels, the circulation is stopped, and the pulp dies from strangulation.

We can readily see that while this is going on, the whole of the pulp cavity being filled by the pulp, the pressure against its walls would be such as to cause intense pain. To guard against this, I always make a free exposure of the pulp before making an application. This gives an opening or vent for relief from the increased pressure. I have used but one thing to destroy the pulp for many years, and that is arsenic. I am aware that many decry it, and attribute most pernicious effects to its use, but so far I know of nothing better, and shall be most happy to change when I do. But I do not use the arsenic of commerce, which, notwithstanding its cheapness, is largely adultered. I use pure crystal arsenic, and the quantity required is so small that a piece the size of the one I now hold in my hand ought to last an honest dentist ten years or more.

I make a very free exposure of the pulp, for by this means I am able to see it. I do not believe in filling teeth by faith, though we are told in the 10th chapter of Timothy, 16th verse, that "faith is the substance of things hoped for." That is all very well; we can *hope* for anything under heaven, but we can not *expect* to accomplish any good thing unless we start right and work right, and to do this it is necessary to see what we are doing.

Having made a free opening, I touch the exposed portion with pure carbolic acid, then take my piece of crystal arsenic, and with a knife or instrument scrape a very little of it into powder. I twist a piece of cotton the size of a pin-head on a smooth nerve instrument, so loosely that it is easily detached; moisten with carbolic acid and take up as much of the arsenic as will adhere to it, and place it upon the exposed pulp, but without any pressure. I then place a small concave metal disk over it, in such a manner that it rests upon the walls of the cavity, and effectually prevents any pressure upon the pulp I then fill carefully with gutta-percha, and if there is no pressure there will be no pain. If there is pressure you will hear from it at once, which means to remove and start again.

These disks or caps are about one-eighth of an inch in diameter, and I usually make a quantity by punching them from the thin sheet iron that instrument makers mail instruments in. Do not, to save a little trouble and time, cover the arsenic with cotton dipped in sandarac instead of gutta-percha. It is a solvenly way to work, and it is not safe. I have seen many cases in which the arsenic had escaped from the tooth, destroying the alveolar septum, and not infrequently causing the loss of the tooth itself. In most such cases the dressing had been cotton and sandarac.

I prefer to allow the arsenic to remain five, six or seven days, as then the pulp can generally be removed whole. It does not break and tear, as it will where it has only been allowed twenty-four hours. It is quite tough and leathery, and can be removed with no pain, except where it separates at the foramen. If after three or four days the tooth becomes sensitive to the touch, remove the filling, cap, etc., and it will return to its normal condition in a day or two, when the pulp can be removed. All this takes time, and time is money to a dentist if it is to any one; but take all the time necessary to do work well, and do not forget to charge well for it, for its value can not be overestimated.—*Western Dental Journal.*

